# The nontoxic natural compound Curcumin exerts anti-proliferative, anti-migratory, and anti-invasive properties against malignant gliomas

**DOI:** 10.1186/1471-2407-10-491

**Published:** 2010-09-14

**Authors:** Christian Senft, Margareth Polacin, Maike Priester, Volker Seifert, Donat Kögel, Jakob Weissenberger

**Affiliations:** 1 Department of Neurosurgery, Goethe-University, Schleusenweg 2-16, 60528 Frankfurt, Germany; 2 Division of Experimental Neurosurgery, Goethe-University, Heinrich-Hoffmann-Str. 8, 60528 Frankfurt, Germany

## Abstract

**Background:**

New drugs are constantly sought after to improve the survival of patients with malignant gliomas. The ideal substance would selectively target tumor cells without eliciting toxic side effects. Here, we report on the anti-proliferative, anti-migratory, and anti-invasive properties of the natural, nontoxic compound Curcumin observed in five human glioblastoma (GBM) cell lines *in vitro*.

**Methods:**

We used monolayer wound healing assays, modified Boyden chamber trans-well assays, and cell growth assays to quantify cell migration, invasion, and proliferation in the absence or presence of Curcumin at various concentrations. Levels of the transcription factor phospho-STAT3, a potential target of Curcumin, were determined by sandwich-ELISA. Subsequent effects on transcription of genes regulating the cell cycle were analyzed by quantitative real-time PCR. Effects on apoptosis were determined by caspase assays.

**Results:**

Curcumin potently inhibited GBM cell proliferation as well as migration and invasion in all cell lines contingent on dose. Simultaneously, levels of the biologically active phospho-STAT3 were decreased and correlated with reduced transcription of the cell cycle regulating gene c-Myc and proliferation marking Ki-67, pointing to a potential mechanism by which Curcumin slows tumor growth.

**Conclusions:**

Curcumin is part of the diet of millions of people every day and is without known toxic side effects. Our data show that Curcumin bears anti-proliferative, anti-migratory, and anti-invasive properties against GBM cells *in vitro*. These results warrant further *in vivo *analyses and indicate a potential role of Curcumin in the treatment of malignant gliomas.

## Background

Although the introduction of temozolomide treatment in addition to radiotherapy after surgical resection has improved survival in patients with glioblastoma (GBM), tumor recurrence is inevitable [1,2]. After tumor recurrence, current as well as novel chemotherapeutic regimens are of modest benefit, and overall survival rates remain poor [3]. Only a subpopulation of patients (with a methylated O(6)-methylguanine-DNA methyltransferase (MGMT) gene promoter) may benefit from dose-intensified temozolomide treatment with added lomustine in terms of overall survival, at the cost of increased toxicity [4]. Therefore, new drugs that are effective in a wider range of GBM patients, most preferably without inducing additional toxicity, continue to be sought.

Curcumin, derived from the rhizome of the plant *Curcuma longa*, is the major pharmacologically active component of the spice turmeric and potentially represents one of those drugs [5]. Being the main ingredient of curries and thus part of the everyday diet of millions of people, Curcumin is considered a safe agent in humans [5,6]. Recent preclinical as well as first clinical reports have indicated that Curcumin may be effective in the treatment of various cancers [7-10]. The underlying mechanisms of this efficacy are still under investigation, but recently an association with the JAK/STAT3 pathway has been proposed [11].

With this study, we aimed to assess the potential effects of treatment with Curcumin on the hallmarks of GBM, i.e. tumor cell proliferation, migration, and invasion and to investigate the potential mechanisms of action.

## Methods

### Cell culture

Cell lines studied were derived from human primary (A-172, MZ-18) or recurrent GBM (MZ-54, MZ-256, MZ-304) and grown in high glucose (4.5 g/l) DMEM with 10% heat inactivated fetal calf serum (FCS), 100 U/ml penicillin, and 100 mg/ml streptomycin. Cells were cultured at 37°C in a humidified atmosphere composed of 5% CO_2 _and 95% air.

### Chemical reagents

Curcumin (94% pure) and 3-(4,5-dimethylthiazol-2-yl)-2,5-diphenyl tetrazolium bromide (MTT) were purchased from LKT (LKT laboratories, St. Paul, MN, USA) and *Sigma-Aldrich (Sigma-Aldrich Chemie GmbH, Taufkirchen, Germany*), respectively. For stock solutions, Curcumin was dissolved in DMSO at 10 mg/mL and stored at -20°C; MTT was dissolved in PBS at 5 mg/mL and stored at 4°C.

### Cell growth and proliferation assay

Cell viability was determined using the methyl-thiazolyl tetrazolium bromide (MTT) quantitative colorimetric assay. The viable cell number is directly proportional to the production of insoluble purple formazan through cleavage of the tetrazolium ring by mitochondrial enzymes. The coversion can be measured spectrophotometrically (λ = 560 nm) upon solubilization with 1/24 1 M HCl/95% EtOH.

Cells were seeded at a density of 5,000 cells/well in a 96-well-plate (Greiner Bio-One, Frickenhausen, Germany) and were allowed to grow in medium containing 10% FCS for 24 hours. Thereafter, cells were incubated with Curcumin at concentrations of 0, 10, 20, and 50 μM. Cells were allowed to grow for various periods of time (6, 12, 24, 48, and 72 hours). Thereafter, cells were incubated with MTT (0.5 mg/ml) for 3 hours. Cell growth was determined by measuring absorption at indicated periods of time using a multi-well scanning reader (Tecan GmbH, Crailsheim, Germany). For each experiment, 18 wells were allocated to one treatment or control group.

### Wound healing assay

Monolayer wound healing assays, a.k.a. scratch assays, were performed by plating cells in 6-well culture dishes (Greiner Bio-One, Frickenhausen, Germany) as described previously [12].

Briefly, 15 - 20 × 10^5 ^cells were seeded per well. After the cells were allowed to attach and reach 80% subconfluency, they were incubated with starvation medium containing 2% FCS for 24 hours prior to further incubation for 2 hours in starvation medium in the absence (control) or presence of Curcumin at concentrations of 10, 20, and 50 μM, before a scratch was performed through the cell monolayer using a yellow pipet tip. Cells were washed with PBS before photographs of the scratch area were taken in treated and untreated cells using a Nikon Eclipse TE2000-S microscope (Nikon GmbH, Düsseldorf, Germany). For each well, two different areas of the scratch were photographed and their location on the dish was noted. Cells were further incubated for 12 hours in starvation medium before the exact same areas were re-photographed and cells entering the denuded area were counted.

### Invasion assay

Invasion of tumor cells was evaluated using a Matrigel-coated modified Boyden chamber (Biocoat™ Matrigel™ Invasion Chamber; Becton Dickinson GmbH, Heidelberg, Germany) according to the manufacturer's advice.

Briefly, 25,000 cells untreated or treated with Curcumin at concentrations of 10 and 20 μM were seeded into the upper well of the chamber containing serum-free culture medium. The lower well was filled with culture medium containing 10% FCS. After 24 hours cells on the upper surface of the well were removed and cells on the lower surface were fixed in 95% ethanol and stained with 0.1% crystal violet. Then, the transmigrated cells were counted using a Nikon Eclipse TE2000-S microscope (Nikon GmbH, Düsseldorf, Germany). For each experiment, 10 random high power fields were counted.

### Sandwich ELISA

To elucidate the potential mechanism of action, we examined the effect of Curcumin treatment on the phosphorylation status of the transcription factor STAT3 employing a sandwich-ELISA kit (PathScan^® ^Phospho-Stat3 (Tyr705) Sandwich ELISA Antibody Pair #7146; Cell Signaling Technology Inc., Danvers, MA) according to the manufacturer's advice.

Briefly, after coating the microplate wells, cells were seeded on 10 cm Ø culture dishes and were incubated for 2 h with Curcumin at 0, 10, 20, or 50 μM, respectively. Cells were then lysed using ice-cold lysis buffer; the lysates were further sonicated on ice. Then, 100 μl of the respective lysates were added to a microplate well and incubated at 37°C for 2 h before the well was washed, and first a detection antibody (incubation: 1 h) and then a secondary antibody (incubation: 30 min) was added to each well. After finally adding TMB substrate and STOP solution, absorbance of each well was measured at λ = 450 nm.

### Quantitative *real-time *PCR

The quantification of mRNA levels was carried out using a *real-time *fluorescence detection method (TaqMan^®^) as described previously [13]. Quantitative *real-time *PCR plots the PCR product on a curve as it accumulates at each cycle of the reaction, in contrast to conventional PCR, which only displays PCR product at the final cycle. Total RNA was reversely transcribed using *SuperScript*™ *III *reverse transcriptase (Invitrogen GmbH, Karlsruhe, Germany). Subsequently, approximately 30 ng of cDNA were subjected to amplification using an ABI Prism 7500 sequence detection system with TaqMan^® ^assays (Applied Biosystems Inc, Foster City, CA) according to the manufacturer's advice. Primers and probes were designed to specifically amplify mRNA of c-Myc (Hs01032443_m1 and Hs00905030_m1), *Ki-67 *(Hs00153408_m1 and Hs01032435_g1) as well as mRNA of a reference gene, HPRT-1 (Hs99999909_m1). The ratios of c-Myc or *Ki-67 *RNA to the reference HPRT-1 represent their relative expression levels. Expression changes were analyzed with the 2-^ΔΔCt ^method [14].

### Caspase cleavage assay

Effector caspase activity of treated and untreated cells was determined as described previously [15]. Briefly, buffer containing DEVD-7-amino-4-methylcoumarin (AMC) was added to the lysates of treated (24 h) and untreated cells at a final concentration of 10 μmol/L. Cells treated with staurosporine (STS) at 3 μM for 16 h served as control. Cells were incubated for 2 h at 37°C in the dark and the generation of the fluorescent AMC cleavage product was measured at 380 nm excitation and 465 nm emission, using a fluorescence plate reader. Fluorescence of blanks containing no cell lysate was subtracted from the values. Protein content was determined using the Pierce Coomassie Plus Protein Assay reagent (KMF, Cologne, Germany). Caspase activity is expressed as change in fluorescence units per microgram protein per hour.

### Statistical analysis

All data are expressed as means ± standard error of the mean (SEM) of at least 3 independent experiments. Statistical differences were evaluated by 1-way ANOVA followed by Tukey's test using commercially available software (SPSS 17.0; SPSS Inc., Chicago, Ill.). P values < 0.05 were considered statistically significant.

## Results

### Curcumin is a potent inhibitor of GBM proliferation

**Figure 1:**
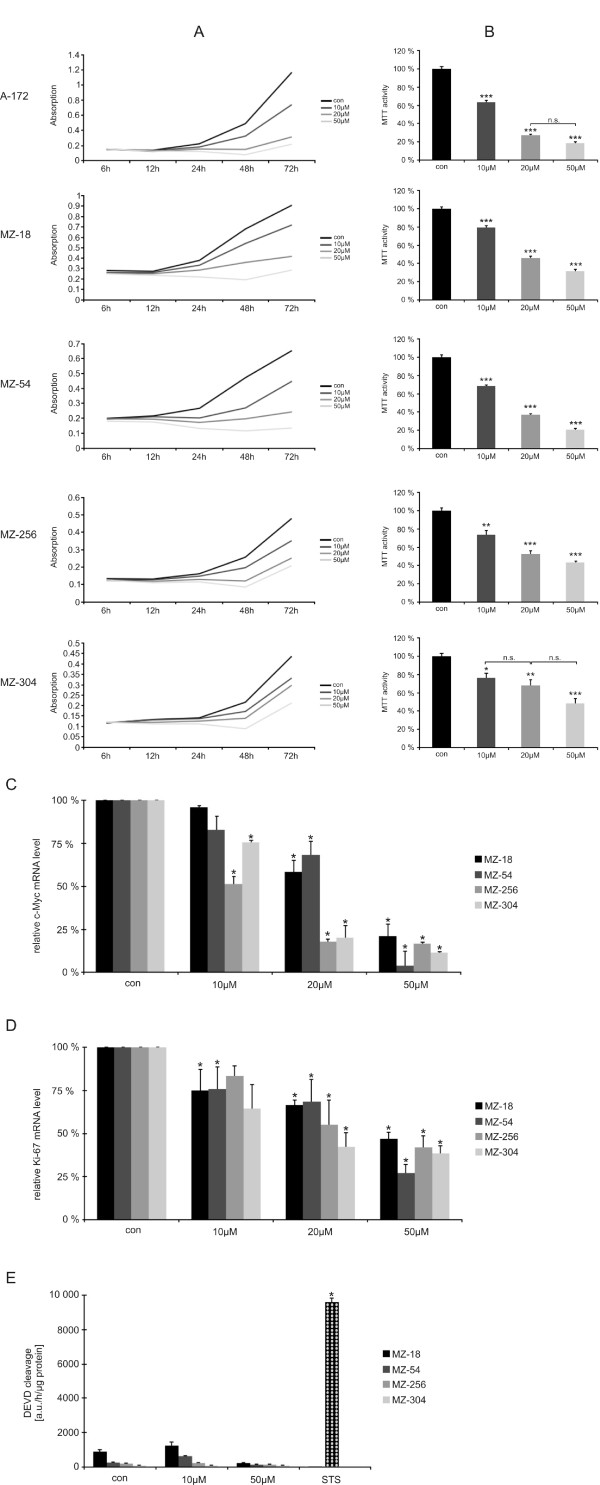
**Cell proliferation**. **A**. Line graphs showing representative growth curves of human GBM cells when treated with or without Curcumin at various concentrations (10 μM, 20 μM, or 50 μM, respectively). **B**. Bar graphs showing dose-dependency of cell viability when treated with Curcumin (0, 10 μM, 20 μM, or 50 μM, respectively) after 72 h. Data are from three independent experiments. Controls are set at 100%. Statistically significant differences compared to controls are marked by asterisks (* P < 0.05, ** P < 0.01, *** P < 0.001). Unless indicated by n.s., differences between groups are statistically significant (P < 0.05 or less). **C**. Bar graphs showing decrease in genomic transcription of c-Myc after treatment with Curcumin (0, 10 μM, 20 μM, or 50 μM, respectively) for 2 h. Data are from three independent experiments. Controls are set at 100%. An asterisk indicates differences that are statistically significant compared to controls. **D**. Bar graphs showing decrease in genomic transcription of Ki-67 after treatment with Curcumin (0, 10 μM, 20 μM, or 50 μM, respectively) for 24 h. Data are from three independent experiments. Controls are set at 100%. An asterisk indicates differences that are statistically significant compared to controls. **E**. Bar graphs showing effect of treatment with Curcumin (0, 10 μM, 20 μM, or 50 μM, respectively) for 24 h on caspase 3-like activity. Data are from three independent experiments. Staurosporine (STS) treated cells served as a positive control for induction of apoptosis. An asterisk indicates differences that are statistically significant compared to untreated cells.

To examine whether treatment with Curcumin influences tumor cell proliferation, we employed MTT assays. In a dose-dependent fashion, cell growth was reduced in all cell lines as shown by cell proliferation graphs depicted in Figure 1A. Already, low dose (10 μM) treatment with Curcumin significantly reduced cell growth after 72 h by 21% - 36% (MZ-18 and A-172, respectively). An even stronger effect was observed after incubation with 20 or 50 μM Curcumin, reducing cell growth by at least 32% (20 μM, MZ-304) to 81% (50 μM, A-172). Details are provided in Figure 1B.

### Curcumin reduces intracellular levels of the transcription factor STAT3, resulting in reduced transcription of cell cycle regulating genes

**Figure 2:**
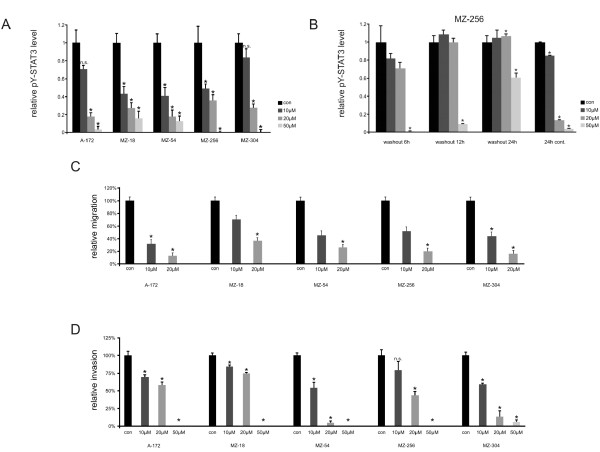
**Cell migration/invasion and STAT3 levels**. **A**. Bar graphs showing a dose-dependent reduction of intracellular levels of phosphorylated STAT3 by Curcumin (0, 10 μM, 20 μM, or 50 μM, respectively) determined by ELISA. Data are from four independent experiments. An asterisk indicates differences that are statistically significant compared to controls. **B**. Bar graphs showing restoration of phosphorylated STAT3 levels in MZ-256 cells following treatment with Curcumin (0, 10, 20, 50 μM) for 2 h as determined by sandwich ELISA. In cells treated with 10 or 20 μM, normal levels are restored after 12 h, and they further increase after 24 h. Phosphorylated STAT3 levels remain low for up to 24 h in cells treated with 50 μM Curcumin. When in contrast cells are treated with Curcumin continuously for 24 h, phosphorylated STAT3 levels remain low. Data are from three independent experiments. An asterisk indicates differences that are statistically significant compared to controls. **C**. Bar graphs showing a dose-dependent reduction of GBM cell motility by Curcumin (0, 10 μM, or 20 μM, respectively) determined by wound healing assays. Data are from three independent experiments. An asterisk indicates differences that are statistically significant compared to controls. **D**. Bar graphs showing a dose-dependent reduction of the invasive capability of GBM cells by Curcumin (0, 10 μM, 20 μM, or 50 μM, respectively) determined by modified Boyden chamber assays. Data are from three independent experiments. An asterisk indicates differences that are statistically significant compared to controls.

We hypothesized that the effects on cell proliferation induced by Curcumin may be explained by its interference with the JAK/STAT3-pathway, as Curcumin was shown to activate the tyrosine phosphatase SHP-2, a negative regulator of JAK activity [16]. STAT3, activated by JAKs, is a nuclear transcription factor, known to regulate genes involved in cell cycle progression [17]. We previously reported that STAT3 is constitutively activated in the cell lines used [18]. In parallel to our observation of reduced cell proliferation, we found reduced transcription of cell cycle regulating c-Myc already after 2 h of Curcumin treatment (Figure 1C). Correspondingly, quantitative *real-time *PCR also revealed a decrease of Ki-67 mRNA synthesis after 24 h incubation with Curcumin (Figure 1D). In concordance with the reduced transcription of cell cycle regulating genes, we observed a dose-dependent reduction of phosphorylated (active) STAT3 levels after 2 h treatment with Curcumin in all cell lines investigated as determined by ELISA. When normalized to untreated controls, phospho-STAT3 levels declined to 41-83% after treatment with 10 μM Curcumin and to 18-35% after treatment with 20 μM Curcumin. Phospho-STAT3 levels eventually diminished to 0-16% after treatment with 50 μM Curcumin (Figure 2A).

To examine whether STAT3 inhibition by Curcumin is short-lived or long-lasting, we additionally performed wash out experiments with MZ-256 GBM cells. As indicated in Figure 2B, the continuous presence of 50 μM Curcumin decreased STAT3 tyrosine-705 phosphorylation completely for over 24 h, while after withdrawal of the inhibitor the active form of the transcription factor STAT3 began to resurface at 12 h after the wash out to reach 60% of its control level after 24 h. This experiment revealed that the estimated half-life of Curcumin in cultured GBM cells is about 24 h. It can therefore be concluded that STAT3 inhibition by Curcumin is transient, and Curcumin has to be sustained continuously for effective treatment.

### Curcumin inhibits GBM migration and invasion

Having established a link between Curcumin and phospho-STAT3, we further investigated the effect of Curcumin on the migratory behavior of GBM cells by performing wound healing assays. Here, we found that Curcumin treatment significantly inhibited cell migration in all cell lines in a dose-dependent fashion (Figure 2B). In addition, we performed trans-well assays using modified Boyden chambers to investigate the effects of Curcumin on the invasive properties of GBM cells. Our findings here were comparable to the wound healing assays with a dramatically reduced invasiveness of cells after treatment with Curcumin. At a concentration of 50 μM Curcumin, only in the MZ-304 cell line there were a few cells invading trough the matrigel membrane; in all other cell lines, the capability to invade the membrane was completely abolished (Figure 2C).

### Effect of Curcumin on apoptosis in GBM cells

To investigate whether curcumin may not only inhibit cell proliferation, but also induce apoptosis in GBM cells, a caspase 3-like DEVD cleavage assay was employed with staurosporine (STS) serving as a positive control for induction of apoptosis. After treatment with Curcumin, we observed neglibigle induction of effector caspases, whereas STS induced significant DEVD cleavage activity (Figure 1E).

## Discussion

Until today, glioblastomas are incurable malignant tumors. Neither the implementation of multimodal therapies nor advances in surgical techniques have helped to push median survival of affected patients above the 2-year boundary [1,19,20]. Therefore, new therapeutic strategies are constantly under investigation. Ideally, a chemotherapeutic drug would prove efficacious selectively against tumor cells without inducing unwanted side effects.

Although long-term studies in both animals and humans are lacking, Curcumin, being a natural compound and the main ingredient of turmeric, commonly known as "curry", is generally regarded as a safe agent [21]. Therapeutic effects on various cancers have been reported [7,22]. Besides showing an inherent cytotoxicity against malignant cells, Curcumin has additionally been shown to modulate radio- and chemosensitivity of cancer cells [10,23-25]. With regards to its potential anti-cancer properties, epidemiological data show a generally low incidence in several types of cancer in populations consuming around 100-200 mg/day [26]. A recent phase I clinical trial in breast cancer demonstrated safety of a daily intake of 6-8 g Curcumin [27]. Several molecular targets of Curcumin have been implicated in the anticancer effects of Curcumin, and Curcumin was suggested to affect a number of molecular signaling cascades [21,28,29].

In this study, we could show that Curcumin potently inhibits proliferation of GBM cells. Our data further indicate that the efficacy of Curcumin can be explained by interference with the JAK/STAT3-pathway. STAT3 inhibition represents a novel target in the treatment of brain tumors. In its active form, STAT3 regulates a number of pathways important in tumorigenesis including cell cycle progression, migration, and invasion [30]. In gliomas, there are several reports on a constitutive activation of STAT3 [31]. Normal cells, in contrast to tumor cells are relatively tolerant to interruption of the STAT3 signaling pathway, making STAT3 an excellent target for molecular therapy of cancer [32,33]. Gliomas seem to depend on activated STAT3: inhibition of STAT3 is known to suppress proliferation [34], and STAT3 knockdown reportedly induces apoptosis in glioma cells [35]. Inhibition of STAT3 also leads to reduced transcription of cell cycle regulating genes such as c-Myc [30]. Here, we demonstrate that Curcumin reduces intracellular levels of biologically active phosphorylated STAT3 in all GBM cell lines used contingent on dose, which is paralleled by reduced transcription of c-Myc and Ki-67. Thus, our data indicate that the effect of Curcumin on GBM proliferation is mediated through interference with the STAT3 signaling pathway. This conclusion is in line with previous observations in other cancers [36,37].

We did not observe significant induction of apoptosis in our caspase assays. Therefore, the robust antiproliferative effects of Curcumin as measured in the MTT assays indeed reflect an inhibition of cell growth and were not caused by an overall cell loss due to apoptosis in the cultures. This finding is in line with previous reports demonstrating cell cycle arrest caused by Curcumin [38].

In addition to cell growth, treatment with Curcumin affected another hallmark of gliomas, i.e. migration and invasion. We could recently demonstrate that interference with the JAK/STAT3 pathway inhibits genomic transcription of MMPs and results in decreased proteolytic activity of MMPs 2 and 9 affecting GBM migration and invasion [18]. Yet, in another report Curcumin inhibited MMP gene expression through interference with the MAP kinase pathway [39]. It is therefore possible, that the effects of Curcumin could partially be exerted through several different molecular targets. Due to the variety of potential interactions, it cannot be ruled out that the observed anti-proliferative effect of Curcumin might be exerted by interference with another pathway in addition to JAK/STAT3. However, our study strongly supports the hypothesis that STAT3 is one of the key targets of Curcumin [36,40]. Likewise, several other groups have reported STAT3 to be associated with migration and invasion in glial as well as non-glial tumors [41,42]. Finally, STAT3 was most recently considered to be a master regulator of human gliomas and essential for maintaining tumor initiating capacity and ability to invade the normal brain [43].

We have shown here that Curcumin potently hampers GBM cell proliferation, migration, and invasion, and our data suggest that this effect is mediated through interference with the JAK/STAT3 pathway. Given the fact that STAT3 plays a key role in the mesenchymal transformation of gliomas, which accompanies aggressive behavior [43], STAT3 may also be a prime target to prevent malignant transformation of low-grade gliomas. Our data, along with existing reports in the literature, indicate that Curcumin could become part of the therapeutic armamentarium in the multimodal treatment of glioma patients. So far, Curcumin represents a safe and low-cost drug, whose application in clinical practice, even in high doses, in addition to conventional chemotherapeutics is under investigation in early phase clinical cancer trials [27]. In the future, experimental as well as clinical studies e.g. regarding the combination of Curcumin and temozolomide or Curcumin and radiation therapy will further elucidate its therapeutic value in malignant gliomas.

## Conclusions

Our data suggest that Curcumin is an effective agent to target GBM cell proliferation as well as migration and invasion. Its effects are at least partially mediated by interference with the STAT3 signaling pathway. Exerting anti-tumor properties without inducing toxicity, Curcumin represents a promising agent against GBM and other cancers. Further analyses are warranted and necessary to substantiate our findings.

## Competing interests

The authors declare that they have no competing interests.

## Authors' contributions

CS conceived the study, performed MTT assays, migration assays, invasion assays, PCR, and ELISAs, analyzed the data, and drafted the manuscript. MPo performed cell culture and participated in MTT assays and real-time PCR. MPr performed cell culture and caspase assays, and participated in migration assays. VS, DK, and JW conceived the study, supervised the experiments, and helped to draft the manuscript. All authors have read and approved the manuscript.

## Pre-publication history

The pre-publication history for this paper can be accessed here:

http://www.biomedcentral.com/1471-2407/10/491/prepub
